# Cerebral glycolysis: a century of persistent misunderstanding and misconception

**DOI:** 10.3389/fnins.2014.00360

**Published:** 2014-11-19

**Authors:** Avital Schurr

**Affiliations:** Department of Anesthesiology and Perioperative Medicine, University of Louisville School of MedicineLouisville, KY, USA

**Keywords:** cerebral energy metabolism, glycolysis, lactate, mitochondrial LDH, NAD-NADH recycling, habit of mind

## Abstract

Since its discovery in 1780, lactate (lactic acid) has been blamed for almost any illness outcome in which its levels are elevated. Beginning in the mid-1980s, studies on both muscle and brain tissues, have suggested that lactate plays a role in bioenergetics. However, great skepticism and, at times, outright antagonism has been exhibited by many to any perceived role for this monocarboxylate in energy metabolism. The present review attempts to trace the negative attitudes about lactate to the first four or five decades of research on carbohydrate metabolism and its dogma according to which lactate is a useless anaerobic end-product of glycolysis. The main thrust here is the review of dozens of scientific publications, many by the leading scientists of their times, through the first half of the twentieth century. Consequently, it is concluded that there exists a barrier, described by Howard Margolis as “habit of mind,” that many scientists find impossible to cross. The term suggests “*entrenched responses that ordinarily occur without conscious attention and that, even if noticed, are hard to change*.” Habit of mind has undoubtedly played a major role in the above mentioned negative attitudes toward lactate. As early as the 1920s, scientists investigating brain carbohydrate metabolism had discovered that lactate can be oxidized by brain tissue preparations, yet their own habit of mind redirected them to believe that such an oxidation is simply a disposal mechanism of this “poisonous” compound. The last section of the review invites the reader to consider a postulated alternative glycolytic pathway in cerebral and, possibly, in most other tissues, where no distinction is being made between aerobic and anaerobic glycolysis; lactate is always the glycolytic end product. Aerobically, lactate is readily shuttled and transported into the mitochondrion, where it is converted to pyruvate via a mitochondrial lactate dehydrogenase (mLDH) and then is entered the tricarboxylic acid (TCA) cycle.

## Introduction

More than 70 years ago, the identity and sequence of the reactions of glycolysis, also known as the *Embden-Meyerhof pathway*, were elucidated. Nevertheless, for the past 25 years investigators in the field of brain energy metabolism have been hotly debating the details of that sequence. A somewhat similar debate first took place among exercise physiologists and biochemists when Brooks (1985) published results showing that lactic acid (lactate) is the glycolytic product and the oxidative substrate during sustained exercise. Soon thereafter, a few studies by neuroscientists questioned the status quo in our understanding of how the brain handles increased energy requirements during stimulation. First, Fox and Raichle (1986) demonstrated a focal physiological uncoupling between cerebral blood flow and oxidative metabolism upon somatosensory stimulation in humans. Two years later Fox et al. (1988) showed that during focal physiologic neural activity the consumption of glucose is non-oxidative. Simultaneously, Schurr et al. (1988) demonstrated the ability of brain (hippocampal) slices to maintain normal synaptic function with lactate as the sole oxidative energy substrate. Many scientists in the field were surprised by these findings, while others discounted them (Chih et al., 2001; Dienel and Hertz, 2001; Chih and Roberts, 2003; Dienel and Cruz, 2004; Hertz, 2004; Fillenz, 2005). Despite the allowance of time necessary for new findings to overcome “habits of mind” (Margolis, 1993) or the incommensurability of “new” and “old” paradigms (Kuhn, 1996), the great debate has not subsided. Hence, lines have been drawn between two camps; one, still a majority, which discounts any key role for lactate in brain (and muscle) energy metabolism and another, a growing minority, which holds lactate as an important, and at times, crucial, oxidative substrate for energy production in the brain (and other tissues).

The unusual longevity of this debate is somewhat surprising. Being on the minority side of it, I have been intrigued by both its persistence and its emotional flair. The drive to settle the unresolved issues that continue to sustain this debate has prompted the following review of the recorded research on energy metabolism through the formative years of the field of biochemistry during the first half of the twentieth century. The aim of this review has been to uncover the basis and reasoning for lactate's long-lasting negative reputation among scientists and clinicians that has prevented its “rehabilitation” and thus its consideration as an integral part of oxidative energy metabolism. Suspicions that lactate's ill reputation has contributed greatly to its dismissal as anything, but useless end-product of anaerobic energy metabolism, led me to search for recorded hints to discount any such suspicions. Upon reading through the troves of research papers of the past, it is clear that one cannot separate the science from the scientists who practice it. Disagreements among investigators in the fields of muscle and brain energy metabolism had already existed in the early decades of the twentieth century. Lactate, by the majority of interpretations of research results, had been considered for a long time to be a product that must be disposed of in order to achieve normalization of tissue functioning. This is despite findings by several investigators of brain energy metabolism in the 1920 and 1930s, who demonstrated the ability, especially of brain gray matter, to oxidize lactate. With an emphasis on glycolysis, this paper attempts to sort out as many as possible conceptions and misconceptions about (brain) energy metabolism in the formative years of modern biochemistry. A plausible explanation is proposed for how that great leap in knowledge, which occurred over seven decades ago, and the research that led to it, have shaped minds and beliefs both then and now. Guidance from the wisdom of three philosophers, Barber (1961), Kuhn (1996) and Margolis (1993) has been instrumental in this attempt to understand how scientific concepts and beliefs have determined both the direction and the pace of scientific progress in the field of energy metabolism.

## Despite its deficiencies, the dogma of muscular glycolysis, circa 1900–1940, has remained unchanged and almost unchallenged, even today

As to the discovery of lactic acid, first in milk and then in muscles, the reader is directed to other sources including a recent review by Gladden (2008). However, before focusing on early brain energy metabolism, a close attention must be given to the pioneering research on muscle respiration and metabolism to comprehend its significant influence on how the former has been conducted and understood. The first strike against lactic acid was, of course, its association with sour (spoiled) milk. Upon its discovery in muscle, lactic acid was quickly blamed for muscle fatigue and rigor. Experiments were carried out specifically to test lactic acid effect on muscle respiration and rigidity. For instance, Fletcher (1898) refers in his research to preliminary reports showing “*that weak solutions of lactic acid (0.1–0.25%), upon injection through the blood vessels of a frog caused immediate rigidity of the muscles*” and to the suggestion that the development of lactic acid during survival (post excision) respiration (CO_2_ discharge) periods was the cause of natural rigor mortis. Fletcher found out that any concentration of lactic acid he used (0.05–5.0%) produced rigor mortis in an excised frog *Gastrocnemius* muscle immersed in it. The higher the lactic acid concentration the quicker rigor mortis set in. Fletcher was a thorough investigator who published his studies in great detail. He has shown that the presence of oxygen prolonged the survival of excised muscle and measured the effect of oxygen on the rate of disposal of lactic acid from it as a way to bring the muscle back to a state of irritability (Fletcher and Hopkins, 1907). The opening paragraph of the authors' paper is very revealing as to how lactic acid was perceived then: “*For a generation it has been recognized that there are means available within the body by which the acid products of muscular activity may be disposed of, and there is already a large body of well-known evidence which indicates that this disposal of acid products—whatever the site of it may be—is most efficient when the conditions for oxidative processes are most favorable, and that it is incomplete when these conditions are unfavorable*.” These investigators set out to investigate the muscle's own means for an oxidative control of lactic acid formation and for the alteration or destruction of lactic acid, which has already been formed.

Locke and Rosenheim (1907) investigated the consumption of dextrose (glucose) by the isolated rabbit heart in an atmosphere of oxygen. They found that cardiac muscle when supplied with both dextrose and oxygen did not produce any lactic acid, similar to the findings in skeletal muscle. These investigators recognized that “*the oxygen supply of the heart in our experiments, although by no means so great as that in the intact organism, was doubtless sufficient to prevent the formation of a detectable amount of lactic acid*.” It should already be clear from the above few examples that prevention of lactate formation and/or its disappearance is simply a means to keep both skeletal and cardiac muscles functioning.

Understandably, with the negative reputation of lactic acid, no one would consider it to be anything but an anaerobic poisonous product that must be disposed of to assure the survival of healthy, respiring tissue or organ. Under such circumstances, the idea that a muscle could utilize lactate aerobically for the production of energy could not have any chance to emerge without direct scientific evidence to support it. And thus, the concept of “lactic acid as the culprit” in muscle fatigue and rigor mortis continued to be forwarded (Burridge, 1910), although research by others at the time (Barcroft and Orbeli, 1910) have pointed out that lactic acid is not all “bad news.” The latter authors found lactic acid to be a valuable accessory in tissue respiration as carbonic acid is, i.e., “*when oxygen reaches the capillaries at a low tension, the lactic acid tends to turn the oxygen out of the blood*.” Nevertheless, Feldman and Hill (1911) investigated human oxygen inhalation during hard work and concluded “*that the increased production of lactic acid by the muscles is due to oxygen want, and that oxygen inhalation has a favorable influence, at any rate in part, by lessening the rise of acid concentration*.” Even Hill, who then had just begun his impressive work on the heat production of muscle contracture (Hill, 1910), explained his findings in a following study (Hill, 1911) thusly: “*the presence of O_2_ diminishes the duration of the reaction which gives out heat*,

A+B+C→ABC+Heat.

*Hence we should expect O*_2_ *to be one of the bodies participating in the reaction: for in that case the velocity would be, among other things, proportional to the concentration of free O*_2_ *in the tissue. Thus, by increasing the O*_2_ *tension in the tissue an atmosphere of O*_2_ *would decrease, and similarly an atmosphere of H*_2_ *would increase, the duration of the heat production. In this connexion, the experiments of Fletcher and Hopkins (1907) on the oxidative removal of lactic acid are very suggestive. They found that the presence of O*_2_ *removed lactic acid, and presumably replaced it in its former position in the tissues*.” Hill attempted to explain muscle contraction using physical principles: “*On stimulation therefore certain molecules are thrown into solution, which before stimulation were lightly connected in some physical or chemical way with other bodies, so as to be inactive. The presence of these chemical molecules sets up a tension, possibly at certain colloidal membranes in the fiber: the tension falls again, owing to the diffusion of these chemical molecules into the general free space in the fiber, away from the sensitive membranes; the molecules are then oxidized, or replaced in their original positions, under the action of O*_2_, *with an evolution of heat proportional to the amount of those bodies present*.”

In another study, Fletcher (1911) went after conflicting “evidence” regarding the chemical action involved in the formation of lactic acid in muscle and in other cells. He also justified his efforts since “*it has been urged by several observers in recent years that considerable and continued production of d-lactic acid* (the old nomenclature of L-lactic acid) *maybe found during autolysis (aseptic or antiseptic) of minced or crushed muscle long after the extinction of irritability and destruction of structure*.” Not surprising, “observers” were making a connection between muscle damage, its death and the production of lactic acid. Fletcher concluded from his studies that “*the evidence hitherto produced of an autolytic production of lactic acid by muscle cannot be accepted*.” Somewhat surprising conclusion in Fletcher's study is that “*no glycolytic enzyme leading to lactic acid formation appears to exist in muscle. After the addition of dextrose to intact surviving muscle, or to preparations of disintegrated muscle, no increase of lactic acid is found in the absence of bacteria*.” Peters (1913), using Hill's calorimeter for heat production measurements combined with those of lactic acid production confirmed both Hill's (1911) and Fletcher and Hopkins's (1907) findings, concluding that “*heat production and lactic acid liberation in fatiguing amphibian muscle are extremely intimately connected*.” Later, Fletcher (1913) repeated his amphibian muscle studies with several mammalian muscles, essentially concluding that muscles from both of these sources are similar in their survival respiration and lactic acid production. That very year, Hill (1913) published results on muscle heat production using a newly designed and constructed “*thermo-electric apparatus with which it was possible to estimate very rapidly the rise of temperature of muscle, if necessary to within a millionth of a degree*.” In the summary of his paper Hill suggested “*that the processes of muscular contraction are due to liberation of lactic acid from some precursor, and that the lactic acid increases the tension in some colloidal structure of the tissue: that the lactic acid precursor is rebuilt after the contraction is over in the presence of, and by the use of oxygen, with the evolution of heat: and finally that the heat liberated by the muscle excited in the complete absence of oxygen is due simply to the breakdown of the lactic acid precursor, and is the same in nature as the heat-production of rigor*.”

Again, the conclusion was that lactic acid induces muscle contraction via a physico-chemical process and, if not disposed of, would result in fatigue and rigor mortis. Roaf (1914) employed an electro-chemical method that recorded increases in acidity when muscle contracts. He, too, concluded “*that the increase in acidity is the cause of the shortening of muscle*.” Moreover, using his heat production measurements of muscle contraction, Hill presented calculations and arguments in the Proceedings of the Physiological Society on February 14, 1914 (Hill, 1914) in support of the hypothesis that lactic acid formed in the muscle after activity is not removed by the process of oxidation, but rather by a process of replacement into its previous position (sugar). He thus argued as follows: “*The production of 1 grm of lactic acid is accompanied by the evolution of about 450 calories. Now I have shown that during the recovery processes of muscles in oxygen there is a ‘recovery heat-production’ of about the same order of size as the heat-production occurring in the initial processes of contraction. In the oxidative removal of 1 grm of lactic acid therefore there is a heat-production of about 450 calories. Now, the oxidation of 1 grm of lactic acid leads to heat-production of about 3700 calories, which is about eight times as large as the quantity observed*… *Therefore, apparently the lactic acid is not oxidized but replaced in its previous position under the influence and with the energy of the oxidation, either (a) of a small part of the lactic acid itself, or (b) of some other body. Evidence given elsewhere shows that it must be some other body. The lactic acid therefore is part of the machine and not part of the fuel*.” Hill voiced his position and, eventually, the position of the majority of his colleagues, that lactic acid is not a fuel, since the expected heat-production of its oxidation was much lower than the calculated value of its complete combustion. It is surprising that Hill would argue that if lactate were a fuel, all the energy of its oxidation would be released as heat. In essence, Hill's own measurements that lactic acid oxidation produces only 12% of the expected heat-production should have indicated to him and others that the majority of the energy released from this oxidation, not measured as heat, could indicate controlled utilization and/or possibly a conversion to some other forms of energy. Nevertheless, Hill's and others' prevailing position on “lactic acid is not a fuel” has endured to the present day.

Fletcher and Brown (1914) also looked into the Inogen theory, according to which, the discharge of energy by the muscle cell—and by inference its discharge by any other cell—depends upon the dissociative breakdown of some labile molecule (Inogen). For the breakdown to occur, oxygen takes Inogen's place beforehand in such a manner that upon the dissociation of the molecule the energy yielded is due to combustion, and the final products, carbonic acid and water, represent the result of that combustion. Furthermore, lactic acid, which supposedly also arises from Inogen, was considered to be either another final product or an intermediate product destined to be used in future reconstruction of the Inogen complex. Based on their experimental results Fletcher and Brown concluded that CO_2_ and lactic acid do not originate from a common source. They emphatically asserted “*that in the muscle the respiratory oxidative process yielding CO_2_ as an immediate product has its chief end in the supply of energy for replacing the lactic acid in the molecular position from which stimulation of some kind has displaced it, and there appears to be no reason at present for supposing that the material which is oxidized in the respiratory process is the same as, or related to, the material from which lactic acid appears and into which, as it seems, it may again disappear*.” Hence, the leading investigators in the field held that lactic acid is a separate entity from the one that is oxidized during muscle respiration and which yields energy and CO_2_.

Meanwhile, other investigators were attempting to explain cocaine's racking effects on its users or the devastation of diabetes through the increased tissue production of lactate. Underhill and Black (1912) studied the influence of cocaine on the metabolism of dogs and rabbits receiving daily injections of the drug. With daily doses of cocaine (20 mg/kg), lactic acid excretion in the urine was markedly increased in well-fed animals. The investigators concluded that the increase in lactic acid elimination in the urine is unlikely associated with increased muscular activity induced by the drug. They also stated that “*lactic acid and carbohydrate metabolism are presumably intimately associated although there are indications that lactic acid may at times arise from more than a single antecedent*.” This statement appears to be an attempt to tie lactic acid to another process besides carbohydrate metabolism, one that might be responsible for the effect of cocaine. Where diabetes was concerned, the famous Ringer (1914) stated that “*parallelism exists between the degree of acidosis and the degree of disturbance in the carbohydrate metabolism*.” Considering the fact that at that time the role of insulin was unknown and glycolysis was yet to be elucidated, the repeated use of this kind of statements about lactic acid and acidosis was part of an accepted and, almost expected, vernacular. Interestingly, Ringer theorized that diabetic mechanism involves the inability to form the glucoside bond of glycogen. Marriott (1914) in his quantitative study of blood acidosis in diabetes cited a discussion of diabetic acidosis by Magnus-Levy in John Hopkins Hospital Bulletin (Magnus-Levy, 1911) who expressed the view that the “*acid poisoned animal and the diabetic patient do not die from the acid which has been eliminated in the neutralized state, but from the acid which remains in the body*.”

By 1916, the general consensus had been “*that sugar is utilized by muscle as a source of energy and the main product of its activity is carbon dioxide*” (Tsuji, 1916). Consensus had not yet achieved “*with regard to the origin of lactic acid formed in the tissues. Some authors ascribe it to the disintegration of carbohydrates (glucose), while others suggest that deaminization of amino acids (alanine) is its source*.” Tsuji (1916), a researcher from Kyoto, Japan, working at the Institute of Physiology, University College, England, employed a heart lung preparation in his studies and summarized his findings as followed: “*1. Lactic acid is produced in the circulating blood of the heart lung preparation under conditions approaching normal. 2. These results may indicate that lactic acid is one of the normal metabolites of muscular activity. 3. The formation of lactic acid is increased in poisoning of chloroform and in the presence of deficient supply of oxygen in a heart lung preparation. 4. When the heart beat is accelerated by adding adrenalin or amino-acid (alanine, glycine or ereptone) to the circulating blood, or the heart work is increased by alterations in the blood-pressure, lactic acid is not only not produced, but the lactic acid previously contained in the circulating blood disappears*.” Finding number 4 of Tusji might be the first time that aerobic lactate disappearance by the heart was mentioned, although the author could not, of course, have had any inclination to consider the possibility of oxidative utilization lacking supportive evidence.

As scientists appear to form an understanding of lactate intermediary role in energy metabolism, efforts to assign other “roles” did not subside. Ito (1916) confirmed the accidental finding by his countryman, Tatsukichi Irisawa, of the presence of lactic acid in pus, and went ahead to determine that *d*-lactic acid (an old nomenclature analogous to today's L-lactic acid) is a constant constituent of pus and is distinctly increased by the autolysis of pus.

Although investigations into the possible enzymatic (ferments) nature of glycolysis were pursued as early as the dawn of the twentieth century, doubters and supporters of a glycolytic enzyme system being an integral part of muscle and other tissues questioned each other's findings as late as 1917. Ransom (1910) was able to prepare ferments from frozen plasma capable of converting glucose or glycogen into lactic acid, CO_2_ and alcohol. Moreover, he was able to precipitate the plasma with alcohol-ether and to obtain a powder which was similarly active, although not with the same velocity. Most interestingly was Ransom's statement that “*there is reason for thinking that the production of lactic acid precedes that of carbon dioxide in the process of fermentation in muscle plasma*.” Of course, lactate production that is followed by CO_2_ production could mean lactate oxidation. However, such a language in those days could not be spoken. By 1917, Hoagland and Mansfield reaffirmed the glycolytic properties of muscular tissue, also demonstrating that dead tissue, while capable of glycolysis and lactic acid production, did not produce CO_2_. The prevailing understanding had been that most of the CO_2_ produced during muscle work is due to lactic acid production, which brings about CO_2_ release from bicarbonate in muscle and blood. Moreover, it was believed that the CO_2_ thus produced stayed within the muscle upon and immediately after the muscle work, assuming that this CO_2_, together with the lactic acid, must necessarily remain inside the muscle fiber itself.

Adam (1921), working on oxygen consumption in muscle and nerve, also rejected the “inogen” idea and speculated that “*at the moment of contraction, the muscle fiber must work by drawing on stores of potential energy with the tissue, and it appears that the function of the oxidations is to restore to its normal resting level. The muscle fiber is further so constructed that the demand for replenishment of these stores of potential energy, available for future activity, is automatically supplied: for the activity of the cell leaves behind a condition leading immediately to an accelerated oxidation*.” Adam also speculated that the muscle's “*resting respiration is an index of an anabolic process, compensating, and proceeding at an equal rate with, some such catabolic process as the survival formation of lactic acid, observed to occur in resting tissues at a constant rate*.” Clearly, the prevailing notion was, and still is in some circles today, that the working muscle does it anaerobically, utilizing energy stores (carbohydrates) to contract, while producing lactic acid. Any oxidative process that takes place comes after the initial non-oxidative one, where its main purpose is to replenish the energy stores, thus the repeated efforts that were made to show carbohydrate production from lactate under aerobic conditions. Foster and Moyle (1921) also attempted to find answers to “*the fate, during recovery in oxygen, of the lactic acid formed in the muscle during fatigue or survival*.” They stated the known facts thusly: “*The contraction of muscle is a strictly anaerobic process, and is accompanies by the production of lactic acid. The recovery process is dependent on the presence of oxygen, and is accompanied by the removal of lactic acid*.” These investigators showed carbohydrate production (mainly as glycogen) upon muscle recovery in oxygen and a corresponding decline in lactic acid content.

Hartree and Hill (1922, 1923) were interested in investigating how the lactic acid produced in the working muscle is accommodated within the muscle in addition to the CO_2_ already there, without raising the hydrogen ion concentration that is likely to destroy the muscle colloidal structure. The authors concluded from their experiments that in muscle, as was known for blood, there is a buffer mechanism, which is much more effective than a bicarbonate solution. They, along with Otto Meyerhof, assumed this buffer mechanism to be an alkali-protein salt capable of neutralizing acid. The concept that working muscle produces lactic acid aerobically and that the CO_2_ released in the process is all due to the acid action on bicarbonate in the tissue, still holds today. Holden (1924) who investigated the “respiration substance” (Meyerhof's term for the enzymes responsible for the glycolytic process) of mammalian muscle, showed that this substance is heat labile and in reality is a collection of irreversibly oxidizable substances, although lactic acid is not one of them.

A complicating issue in glycolysis is the relationship between lactate and glycogen in muscle and, eventually, in other tissues, including brain. Otto Meyerhof and Archibald Hill were co-awarded the Nobel Prize in Physiology or Medicine in 1923 for their discovery of the fixed relationship between the consumption of oxygen and the metabolism of lactic acid in the muscle. Although the importance of the conversion of glycogen to lactate in muscle is still under debate today (Shulman and Rothman, 2001), both Meyerhof and Hill, the two most dominating scientists of their time in the field of muscle energy metabolism, had a long-lasting influence on the direction and progress of that field. The next section of this monograph deals with their influence on both the research and the researchers of brain energy metabolism.

By the mid-1920s, “the lactic acid as a trouble maker” had become a “habit of mind” (Margolis, 1993) and the tendency to look for lactate as the culprit in any disorder or abnormal condition was almost a given. Ronzoni et al. (1924) measured lactic acid production during ether anesthesia, since acidosis had been reported to be one of its consequences. These investigators concluded that “*1. Accumulation of lactic acid accounts in a large part for the acidosis of ether anesthesia. 2. Its increase is independent of CO_2_ tension and produces the changes in pH rather than being itself controlled by pH*… *3. Decreased oxygen supply to tissues does not account for its production. 4. The source of lactic acid seems to be the muscle tissue. 5. Production of lactic acid in the muscle, together with loss of phosphate from the muscle, during anesthesia, points to a breakdown of some hexose phosphate, such as the Embden's ‘lactacidogen’*.” These findings disagreed with those of Koehler (1924) who demonstrated that the acidosis during ether anesthesia “*is the summation effect of CO_2_ excess and alkali deficit. The CO_2_ excess is the result of inefficient respiration probably caused by decreased sensitiveness of the respiratory center*.” Koehler et al. (1925) expanded their studies to measure the production of acidosis by anoxemia and concluded that “*anoxemia is fundamentally of an acidotic nature as far as disturbances in the acid-base balance are concerned*.” In essence, the authors continue to argue that, although lactic acid production continues to rise during axonemia, the amounts are relatively small and thus, they “…*do not presume to state what is the nature of the acidity*.” Evans (1925) investigated the role of lactic acid in resting striated muscle. Here are some of his findings: “*Lactic acid rapidly accumulates in plain muscle when this is kept under anaerobic conditions, but scarcely at all when kept in oxygen*” and “*The oxygen usage of resting plain muscle indicates that the recovery process in oxygen is of much the same nature as that in skeletal muscle; actually the fraction oxidized under experimental conditions was about one third. Owing, it is thought, to the errors incidental to the determination of small amounts of glycogen, it has not been possible, up to the present, to demonstrate that lactic acid arises from glycogen, or indeed, from any carbohydrate. In any case the glycogen content of the tissue is small, though, when allowance is made for the possible errors of experiment, perhaps large enough to make it possible that glycogen is the parent substance from which lactic acid is formed*.”

Clearly, despite the recognition by the Nobel committee given to Meyerhof for his work on glycogen and lactate, doubts persisted about this polycarbohydrate or any carbohydrate as a source of lactate. Riegel (1927a) demonstrated that severe blood hemorrhage in dogs caused an increase in blood lactic acid concentration and that the total increase and its duration were dependent upon the extent of the hemorrhage. Riegel also experimented with injecting sodium lactate to dogs and followed its disappearance (Riegel, 1927b). She summarized her findings thusly:

- “*Sodium lactate injected into dogs in large amounts is readily removed from the blood. The removal may be divided into two phases*:
  1. *A rapid decrease in concentration of lactic acid in the blood due to diffusion of lactic acid from the blood to other body fluids*.
  2. *A slower decrease in concentration due to utilization of lactic acid by the tissues*.
- *Injection of sodium lactate causes an immediate decrease in inorganic phosphate in the blood and a delayed rise in the sugar of blood*.
- *The conclusion is drawn that lactic acid injected into the blood is synthesized to lactacidogen and glycogen by a process analogous to removal of lactic acid formed in muscle exercise*.”

Although the author indicated in her summary that part of the decrease in blood lactate after an injection of sodium lactate is due to lactate utilization by tissues, she did not mean to indicate oxidative utilization for the production of energy, but rather to indicate utilization in the synthesis of glycogen. The postulated synthesis of lactacidogen had meant to indicate the formation of a hexose diphosphate from lactate and inorganic phosphate as was suggested at the time by Embden himself.

While the above list of cited papers is just but a part of a much longer list, it does convey the general gist of the principles by which scientists of the day were guided in their attempts to elucidate the chemical reactions of aerobic and anaerobic glycolysis. Central to all these studies is muscle tissue and its glycolytic formation of lactate, always anaerobically and mainly through the breakdown of glycogen and, when aerobic oxidation occurred, only after muscle contraction, its main purpose is to remove the accumulated lactate and the accompanied acidosis, and hence, the lactate's reputation as the “black sheep” of energy metabolism. Scientists who were involved in muscle glycolytic research vastly outnumbered those who researched brain glycolysis. Naturally, most of the published findings on muscle energy metabolism greatly influenced not only how muscle researchers related to the sometimes “outlying” findings of brain researchers, but even more striking is how brain researchers had related to their own findings, always examining and measuring them with a “muscular” yardstick. This was the “affliction” of the small scientific community that investigated cerebral glycolysis in the early years of the twentieth century. That community considered lactate to be a useless end-product that must be rid of via oxidation. This habit of mind (Margolis, 1993) would become abundantly clear as the work of these scientists is reviewed and analyzed in the following section.

## The study of cerebral glycolysis, circa 1900–1940, was greatly influenced by the muscular dogma and is being largely ignored and forgotten today

In a very early paper Hill and Nabarro (1895) compared the exchange of blood-gasses in brain and muscle during and after tonic and clonic epileptic episodes induced by intravenously injecting essential oil of absinthe to the animal (presumably a dog). From those experiments and based on the results comparing oxygen and carbonic acid content in arteries and veins of muscle and brain, the investigators concluded that “*the brain is not a seat of active combustion, and considering the very small increase in CO_2_ in the torcular blood it seems to us very improbable that the temperature of the brain should be perceptibly greater than that of the blood*.”

According to Holmes (1932), who authored the very first review paper on brain and nerve energy metabolism, “*Tashiro was the first worker to show that nerve produced CO_2_ and ammonia during its metabolism*” in 1913. Later, others “*investigated the gaseous metabolism of nerve, and all of these workers are agreed that nerve uses oxygen and produces CO_2_ during rest, and that these processes are intensified during activity*.” By 1921, Adam had shown that not like resting muscle, the sciatic nerve exhibited a very small effect of stimulation on its respiration rate. “*Even tetanising currents of one minute's to half-an-hour's duration gave a very small total effect, if any…*” Nevertheless, work in Hill's laboratory (Gerard et al., 1927) had shown that nerve (the frog sciatic nerve) produces a measurable amount of heat, which increased during activity (electric stimulation) and of a magnitude that agreed with the magnitude of oxygen consumption. Holmes (1932) in his review indicated the fundamental importance of the above findings as a conclusive proof that “*nervous impulse is a chemical affair*.”

Eric G. Holmes had established himself as a leading investigator of brain energy metabolism beginning with a paper he and his wife, Barbara E. Holmes, published in 1925 (Holmes and Holmes, 1925a). That preliminary publication followed “[T]*he work of Warburg, Posener and Negelein in 1924 who showed that brain tissue is capable of converting large amounts of glucose into lactic acid*.” For that preliminary investigation the Holmes compared glucose metabolism of the brain in a normal animal (rabbit) and in an animal suffering from the effects of a convulsive dose of insulin. They summarized their findings as follows: “*… there is no marked change in the amount of reducing substance as a result of insulin administration*.” By “reducing substance” the authors meant carbohydrates. “*The reducing substance of brain is not capable of giving rise to the formation of lactic acid, although in similar conditions, abundance of lactic acid is formed by the brain from added glucose. Determinations of “resting” lactic acid on the brains of normal and of “insulin” rabbits show a greatly reduced lactic acid formation in the latter case. Neither in “normal” nor in “insulin” brains is there an increase in lactic acid formation over the “resting” value after standing or incubation at body p_H_*.” In a follow-up study, Holmes and Holmes (1925b) determined that a fall in brain lactic acid levels of insulin-treated rabbits “*does not occur until the blood-sugar has reached a fairly low level*. They concluded *that the fall in the resting lactic acid content of brain after insulin injection is not due to a direct effect of insulin in promoting increased oxidation of lactic acid, nor to any direct effect of insulin or an accompanying impurity in depressing the production of lactic acid by the brain cells, but is rather caused by the fall in the blood-sugar level, and the resulting shortage of glucose in the brain*.” By 1926, the Holmes published a detailed study in which they measured the levels of both glycogen and lactate in rabbit brains. They found the content of the former to be “*small, and very variable*,” a finding that they speculated could be the outcome of the procedure of brain tissue preparation through which there might be a rapid breakdown of glycogen. “*The lactic acid content of rabbits' brains shows no appreciable rise, nor does the glycogen content show any significant fall, when the chopped tissue is kept at room temperature, or incubated under anaerobic conditions at alkaline p_H_. Under aerobic conditions, lactic acid rapidly disappears from chopped brain, but the glycogen suffers no significant change. It is suggested that the brain depends upon blood sugar, rather than on any other substance which it stores itself, for lactic acid precursor*.” These investigators thus established that glucose is the precursor of lactic acid in the brain and that under aerobic conditions lactic acid content decreases. Further, the Holmes team (1927) also showed that brain's “*lactic acid formed from glucose supplied by the blood and that the values of lactic acid in the brain fall and rise with the blood sugar, both in hypo- and hyper-glycaemic condition*.” In addition, they found that “*the brain tissue of diabetic, like that of normal animals, is capable of converting glucose to lactic acid, and of removing lactic acid under aerobic conditions*.”

By 1929, Ashford and Holmes had delved into investigating the part played by inorganic phosphate in the production of lactic acid from carbohydrate in brain tissue. This followed the studies on muscle and yeast metabolism that had shown the prominent role phosphate plays in carbohydrate metabolism. The investigators were somewhat surprised that their findings did not line up with the role of phosphate in muscle and yeast carbohydrate metabolism. They summarized their study thusly:

“*1. Inorganic phosphate is liberated from brain tissue both anaerobically and aerobically, and in the presence as well as in the absence of glucose. No evidence of hexosephosphate synthesis has been found at any stage in the process of formation of lactic acid, although the tissue is capable to a small extent of performing this synthesis*.

*2. Both phosphate liberation and lactic acid production from glucose by brain tissue are inhibited by sodium fluoride, but, whilst the former is affected only by a high fluoride concentration, the latter is sensitive to very high dilutions of the salt. No quantitative relationship can be traced between the amounts of phosphate and lactic acid which are prevented from appearing by fluoride*.

*3. Lactic acid is freely formed from glucose, even when all available phosphate is immobilized. The velocity of lactic acid formation from glucose is not increased by the replacement of phosphate*.

*4. Much less lactic acid is formed from glycogen than from glucose; the process is inhibited by fluoride and by immobilizing phosphate. It can be restored by replacing phosphate*.

*5. It is concluded that brain tissue possesses two mechanisms of lactic acid formation: one, involving glucose, is quantitatively the more important, and is independent of phosphate; the other is much smaller, involves glycogen, and depends on the availability of phosphate*.” Ashford and Holmes (1929) and Holmes in a followed up study (1930) have thus demonstrated for the first time a correlation between lactic acid disappearance and oxygen consumption i.e., an aerobic utilization of lactate in brain tissue. Moreover, they show the ability of sodium fluoride (NaF) to inhibit the conversion of glucose to lactate and concomitantly to inhibit oxygen consumption, making use of the first known glycolytic inhibitor. Furthermore, Holmes (1930) found out that NaF completely blocked oxygen consumption in the presence of glucose in brain gray matter preparation. However, if glucose was replaced by lactate, no inhibition of oxygen consumption was observed. And thus, Holmes concluded “*that glucose must be converted into lactic acid before it can be oxidized by the gray matter*.” This straight forward conclusion, as will be discussed later, has been ignored now for more than 80 years. Holmes and Ashford (1930) and Ashford and Holmes (1931) have also related to a ratio known as the “Meyerhof quotient,” which was established by Meyerhof in muscle as: *Total lactic acid disappearing/Lactic acid oxidized*. This ratio was determined to have a value of approximately 3, and was used by Meyerhof and colleagues to support a proposal known as the “Meyerhof cycle.” Accordingly, when lactic acid is added to an oxygenated muscle tissue, the amount of lactic acid disappearing is approximately three times greater than the amount of oxygen consumed in the process. That finding led Meyerhof to propose that the extra lactic acid disappearing beyond what could be accounted for by oxygen consumption must be recycled to a carbohydrate. Expecting to confirm the existence of a similar “Meyerhof quotient” in brain to that of muscle, Ashford and Holmes were unable to demonstrate a “Meyerhof quotient” greater than 1 in oxygenated brain tissue, which prompted them to state that “*there is no synthesis of carbohydrate from that portion of lactic acid which disappears but is not accounted for by O_2_ uptake*.” Moreover, they believed that their “*experiments throw doubt on the reality of the alleged ‘Meyerhof cycle’ in the case of cells in which the actual synthesis of carbohydrate has not been demonstrated by chemical estimation*.” In addition, Holmes and Ashford found that the O_2_ uptake in oxygenated brain tissue shaken with lactate in the presence of bicarbonate buffer in an O_2_/CO_2_ atmosphere is greater than in the presence of phosphate buffer and that such uptake increases with increased oxygen tension in both cases. They also related to the “Meyerhof quotient” as the “respiratory quotient” and found its value, both of brain tissue alone and of tissue oxygenated with extra oxygen, to be close to unity, including in the case of brain from animals rendered hypoglycemic by insulin injection. They concluded that lactate oxidation is unlikely to spare the utilization of another substrate.

In as much as these investigators clearly demonstrated the ability of brain tissue to oxidize lactate, it never occurred to them that the monocarboxylate could be an energy substrate in the brain. The influence of the “muscle school” prevented them from considering lactate to be more than just a substance that the brain is able to get rid of via oxidation. The fact that they could not observe a sparing effect of lactate on other substrates such as glucose also prevented them from thinking of lactate as a substrate. Thus, despite the significant differences they observed between muscle and brain tissues, where lactate was concerned, the scientific community in those days did not change its consideration of lactate as a useless by-product of carbohydrate metabolism, if not worse. Nevertheless, in a paper published in 1933, Holmes hinted at the possibility that lactate oxidation could support brain activity. And yet, a year earlier Quastel and Wheatley (1932), published their studies where they measured oxidations of different substrates by different brains using the Barcroft differential manometer. To increase the accuracy of their measurements, they allowed the brain preparation to become greatly depleted of its oxidizable materials before substrates were added. First they found that “*the rate of oxidation of added substrate to the brain varies inversely to the size of the animal*,” a generalization that does not apply to the muscle. More importantly, for the purpose of the present paper, is their finding that “*glucose, sodium lactate and sodium pyruvate at equivalent concentrations are oxidized at approximately the same rate by brain tissue*.” Also, by their estimates, lactate was completely oxidized by brain tissue. The investigators also found the toxin, iodoacetic acid (IAA), to inhibit the oxidation of glucose by brain. Although they could not categorically state that the inhibition of glucose glycolysis by IAA (and by NaF) is “*evidence that glucose necessarily passes through lactic acid for its oxidation to take place*,” they had clearly considered it as a strong possibility, unlike Holmes (1930), who all but concluded just that. Interestingly, Quastel and Wheatley mentioned that oxalate inhibits glucose oxidation, but unlike IAA and NaF, also inhibits the oxidation of lactate. Clearly, these investigators were not privy then to the existence of lactate dehydrogenase (LDH), which is known to be inhibited by oxalate and by its derivative, oxamate (Schurr and Payne, 2007). Dixon (1935) had reproduced the results of Holmes & Ashford and Quastel & Wheatley, detecting no formation of lactic acid from glucose by brain tissue in oxygen. Dixon surmised that if there is any lactate produced under those conditions, it is produced at a rate slow enough to be removed by complete oxidation, and thus, concluded “*that oxygen exerts its sparing effect on glycolysis at some point in the system prior to the formation of lactic acid*.” Again, despite the clear observation that lactic acid is oxidized completely in oxygenated brain tissue, the dogma that such oxidation has only one purpose, i.e., rid the tissue of its presence, has always prevailed. And since very little or no lactic acid formation from glucose was detected under oxygen atmosphere, the interpretation of that outcome has been that oxygen spares the tissue from forming lactic acid glycolytically. Hence, the “habit of mind” (Margolis, 1993) regarding lactate as a useless by-product of anaerobiosis has entrenched itself also in the minds of the scientists who worked with brain tissue, where lactate oxidation was established and where several of them specifically voiced, based on data from their own studies, that for glucose to be oxidized it must be first converted to lactate. Consequently, and against their own observations, these investigators never considered that the ability of the tissue to oxidized lactate could have any other purpose, besides being a mechanism aimed at the removal of lactic acid from the tissue.

## Has habit of mind played a continuous role in misconstruing the glycolytic pathway?

Has there been a chance that lactate oxidation would imply anything else, but the purging mechanism of the monocarboxylate from the tissue? Could any of the investigators working on the glycolytic breakdown of glucose during the first 40 years of the twentieth century, and especially those who worked with brain tissue, had a chance to interpret these reactions differently? Reviewing the history of the research that had led to the elucidation of the sequence of the glycolytic pathway and considering the possible mindsets of the scientists working in the field, then and today, I believe that the answer to these questions would be “no.” By the late 1930s and early 1940s the cumulative work of some of the leading researchers in the field of bioenergetics, including Meyerhof, Embden, Zimmerman, Fisk, and Subbarow, Lohmann, Kiessling, Cori and Cori, Warburg and many others, had already determined that there are two separate types of glycolysis, aerobic and anaerobic. Accordingly, the former ends up with pyruvate, while the latter ends up with lactate. Eventually, the demonstration that brain tissue is able to completely oxidize lactate did not sway Holmes, Ashford, Quastel and other investigators of brain carbohydrate metabolism to consider other purpose(s) of such a reaction, despite their own speculation that for glucose to be fully oxidized this process must proceed via the formation of lactate. They were all guided (misguided) at the time by the dominant habit of mind and fully accepted the dogma held by the investigators who worked on muscle carbohydrate metabolism, according to which, lactate is a useless end-product of anaerobic glycolysis that the tissue must rid itself of by any means possible. Moreover, the publication of the possible sequence of the citric acid cycle, known today as the tricarboxylic acid (TCA) cycle (Krebs and Johnson, 1937a,b,c; Krebs et al., 1938), 3 years prior to the final elucidation of the glycolytic pathway, had probably strengthened and deepened the hold of that dogma. Krebs and Johnson (1937a,c) proposed, alas with a question mark (see below), that the carbohydrate derivative that interacts with oxaloacetate to form citrate in the TCA cycle is pyruvate. Krebs et al. (1938) opened their paper with the following paragraph: “*From experiments reported in a previous paper (Krebs and Johnson, 1937a) we concluded that carbohydrate is oxidized in animal tissues through the following series of reactions*:

Considering the importance of Krebs and Johnson's work, for which the former was awarded the 1953 Nobel Prize in Physiology or Medicine, the suggestion that pyruvate is the glycolytic product entering the TCA cycle had undoubtedly been of great influence on the elucidators of the sequence of the glycolytic pathway. However, in those days, the role of mitochondria in respiration and the fact that the enzymes of the TCA cycle are located in these organelles were still unknown. Moreover, none of these scientists could have known that mitochondria also contain in their membrane the enzyme lactate dehydrogenase (LDH) (Brandt et al., 1987; Brooks et al., 1999b; Hashimoto et al., 2006; Atlante et al., 2007; Schurr and Payne, 2007; Lemire et al., 2008; Passarella et al., 2008; Gallagher et al., 2009; Elustondo et al., 2013; Jacobs et al., 2013), an enzyme that can easily convert lactate to pyruvate.

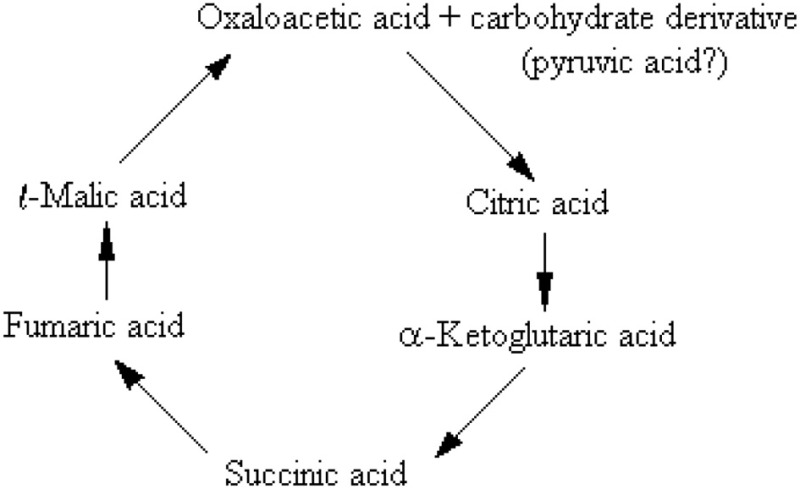



Table 1 lists the references cited and quoted from circa 1900–1940 and summarized their principal findings and interpretations.

**Table 1:** **Circa 1900–1940 cited articles on muscular and cerebral glycolysis: The main findings and their interpretations**.

Hill and Nabarro, 1895—Compared the exchange of blood gasses in brain and muscle during and after epileptic episodes induced by essential oil of absinthe in the dog. Conclusion: The brain is not a seat of active combustion.
Fletcher, 1898—Lactic acid produced rigor mortis in excised frog *Gastrocnemius* muscle when immersed in it. The higher the lactic acid concentration, the faster rigor mortis sets in.
Fletcher and Hopkins, 1907—The body has means to dispose of lactic acid. The most favorable conditions for such a disposal are those that support oxidative processes.
Locke and Rosenheim, 1907—Dextrose and oxygen supply to an isolated rabbit heart are sufficient to prevent any formation of lactic acid, hence better functioning of the heart muscle.
Burridge, 1910—Lactic acid causes muscle fatigue and rigor mortis.
Barcroft and Orbeli, 1910—Lactic acid tends to turn oxygen out of blood capillaries at low oxygen tension, as carbonic acid does, hence it does have some beneficial value.
Feldman and Hill, 1911—The increased lactic acid concentration in the working muscle is due to oxygen want. Oxygen inhalation lessens the rise in acid concentration.
Hill, 1911—The presence of O_2_ in the tissue (muscle) diminishes the duration of heat release of muscle contracture. Thus, by increasing O_2_ tension in the tissue an atmosphere of O_2_ would decrease, and an atmosphere of H_2_ would increase the duration of heat production, suggestive of Fletcher and Hopkins (1907) experiments on the oxidative removal of lactic acid.
Fletcher, 1911—Contrary to observations that there is a connection between muscle damage, its death and production of lactic acid, the author concluded that no glycolytic enzyme leading to lactic acid formation appears to exist in muscle. Addition of dextrose to intact surviving muscle or to preparation of disintegrated muscle did not result in increase lactic acid production in the absence of bacteria.
Underhill and Black, 1912—The increase in lactic acid secretion in the urine of cocaine-treated dogs is unlikely associated with increase muscular activity induced by the drug. Although lactic acid and carbohydrate metabolism are presumably intimately associated, there are indications that lactic acid may arise from more than one source, and thus, possibly be still associated with the effects of cocaine.
Peters, 1913—Agrees with both Hill (1911) and Fletcher and Hopkins (1907) that heat production and lactic acid liberation in fatiguing muscle are extremely intimately connected.
Hill, 1913—The processes of muscular contraction are involved the liberation of lactic acid from some precursor. Lactic acid increases the tension in some colloidal structure of the tissue. The lactic acid precursor is rebuilt and the end of the contraction in the presence of and by the use of O_2_, with the evolution of heat. The heat liberated by excited muscle in the complete absence of O_2_ is due to the breakdown of the lactic acid precursor and is of the same nature of heat production of rigor.
Tashiro, 1913—Was the first investigator to show that nerve produced CO_2_ and ammonia during metabolism.
Roaf, 1914—The increase in acidity is the cause of the shortening of muscle.
Hill, 1914—Argues in support of the hypothesis that lactic acid formed in the muscle after activity is not removed by oxidation, but rather replaced into its previous position, a sugar. Hence, lactic acid is part of the machine, not part of the fuel. A position that endured to present day.
Fletcher and Brown, 1914—Concluded that CO_2_ and lactic acid do not originate from a common source.
Ringer, 1914—About diabetes: Parallelism exists between the degree of acidosis and the degree of disturbance in the carbohydrate metabolism.
Tsuji, 1916—Lactic acid is one of the normal metabolites of muscular activity. By using heart-lung preparation, Tsuji showed that accelerating heart beat via injection of adrenaline or elevated blood pressure the blood concentration of lactic acid declined. That had probably been the first demonstration of heart consumption of lactate an energy substrate, unbeknown to the author.
Ito, 1916—confirmed the accidental finding by his countryman, Tatsukichi Irisawa, of the presence of lactic acid in pus and determined that lactic acid is a constant constituent of pus and is distinctly increased by the autolysis of pus.
Adam, 1921—Concluded that at the moment of contraction, the muscle fiber work by drawing on stores of potential energy within the tissue, and it appears that the function of the oxidations is to restore to its normal resting level. Adam also speculated that the muscle's resting respiration is an index of an anabolic process, compensating, and proceeding at an equal rate with, some such catabolic process as the survival formation of lactic acid, observed to occur in resting tissues at a constant rate.
Foster and Moyle, 1921—Showed that carbohydrate production (mainly as glycogen) occurred upon muscle recovery in oxygen with a corresponding decline in lactic acid content.
Adam, 1921—Showed that not like resting muscle, the sciatic nerve exhibited a very small effect of stimulation on its respiration rate.
Hartree and Hill, 1922, 1923—Concluded from their experiments that in muscle, as was known for blood, there is a buffer mechanism, an alkali-protein salt capable of neutralizing acid, which is much more effective than a bicarbonate solution.
Holden, 1924—Showed that the “respiration substance” (Meyerhof's term for the enzymes responsible for the glycolytic process) of mammalian muscle is heat labile and in reality is a collection of irreversibly oxidizable substances, although lactic acid is not one of them.
Koehler, 1924—Suggested that acidosis during ether anesthesia is the summation effect of CO_2_ excess and alkali deficit. The CO_2_ excess is the result of inefficient respiration probably caused by decreased sensitiveness of the respiratory center.
Ronzoni et al., 1924—Showed that accumulation of lactic acid accounts in a large part for the acidosis of ether anesthesia; that its increase is independent of CO_2_ tension and produces the changes in pH rather than being itself controlled by pH; decreased oxygen supply to tissues does not account for its production; the source of lactic acid seems to be the muscle tissue; production of lactic acid in the muscle, together with loss of phosphate from the muscle, during anesthesia, points to a breakdown of some hexose phosphate.
Koehler et al., 1925—Measured the production of acidosis by anoxemia and concluded that “anoxemia is fundamentally of an acidotic nature as far as disturbances in the acid-base balance are concerned.
Evans, 1925—Showed that lactic acid rapidly accumulates in plain muscle under anaerobic conditions, but scarcely at all when kept in oxygen; that the recovery process in oxygen is of much the same nature as that in skeletal muscle. Suggested that glycogen could be the parent substance from which lactic acid is formed.
Holmes and Holmes, 1925a—Showed that under convulsive dose of insulin there was no marked change in the level of brain reducing substance (carbohydrate) and that this substance in not capable of giving rise to the formation of lactic acid, although and abundance of lactic acid is formed under these conditions from added glucose. Basal levels of lactic acid in “insulin” brains were greatly reduced.
Holmes and Holmes, 1925b—Showed that the fall in brain lactic acid levels of insulin-treated animals does not occur until the blood sugar has reached a fairly low level. Hence, lactic acid levels fall not due to a direct insulin effect, rather to the shortage of glucose.
Holmes and Holmes, 1926—Showed that under aerobic conditions, lactic acid rapidly disappears from chopped brain. Investigator suggested that the brain depends on blood sugar, rather than any other substance which it stores itself, as lactic acid precursor.
Holmes and Holmes, 1927—Showed that the values of brain lactic acid fall and rise with blood sugar, in hypoglycemic and hyperglycemic conditions, respectively.
Riegel, 1927a—Demonstrated that severe blood hemorrhage in dogs caused an increase in blood lactic acid concentration and that the total increase and its duration were dependent upon the extent of the hemorrhage.
Riegel, 1927b—Experimented with injecting sodium lactate to dogs and followed its disappearance. Followed sodium lactate injection blood lactic acid concentration rapidly decreased due to diffusion to other tissues and a slower decrease due to lactic acid utilization by those tissues. She concluded that lactic acid injected into the blood is synthesized to lactacidogen and glycogen by a process analogous to removal of lactic acid formed in muscle exercise.
Gerard et al., 1927—Showed that the frog sciatic nerve produces a measurable amount of heat, which increased during activity (electric stimulation) and of a magnitude that agreed with the magnitude of oxygen consumption, a “conclusive proof that nervous impulse is a chemical affair” (Holmes, 1932).
Ashford and Holmes, 1929; Holmes, 1930—Showed that inorganic phosphate is liberated by brain tissue both anaerobically and aerobically; that phosphate liberation and lactic acid production from glucose by brain tissue are inhibited by NaF, although lactic acid production is much more sensitive to the fluoride; that lactic acid is freely formed from glucose even when all available phosphate is immobilized; that much less lactate is formed from glycogen that from glucose. It was concluded that the brain possesses two mechanisms of lactic acid production: One involves glucose, which is quantitatively more important and is independent of phosphate; the other is much smaller, involves glycogen and depends on phosphate availability. It was also demonstrated, for the first time, that a correlation exists between lactic acid disappearance and oxygen consumption i.e., an aerobic utilization of lactate in brain tissue; the ability of sodium fluoride (NaF) to inhibit the conversion of glucose to lactate and concomitantly to inhibit oxygen consumption, using a glycolytic inhibitor for the first time; in the presence of NaF, oxygen consumption in the presence of glucose was completely blocked in brain gray matter preparation, but by replacing glucose with lactate, fluoride did not inhibit oxygen consumption. Conclusion: Glucose must be converted into lactic acid before it can be oxidized by the gray matter.
Holmes and Ashford, 1930; Ashford and Holmes, 1931—Measured the “Meyerhof quotient,” the [total lactate disappearing]/[lactate oxidize], which in muscle was determined to be ~3, and found it in brain to be 1; found that the O_2_ uptake in oxygenated brain tissue shaken with lactate in the presence of bicarbonate buffer in an O_2_/CO_2_ atmosphere is greater than in the presence of phosphate buffer and that such uptake increases with increased oxygen tension in both cases; termed “Meyerhof quotient” the “respiratory quotient” and found its value, both of brain tissue alone and of tissue oxygenated with extra oxygen, to be close to unity, including in the case of brain from animals rendered hypoglycemic by insulin injection; concluded that lactate oxidation is unlikely to spare the utilization of another substrate.
Quastel and Wheatley, 1932—Found that the rate of oxidation of an added substrate to brain tissue varies inversely with the size of the animal, a generalization that does not apply to muscle; that glucose, lactate and pyruvate at equivalent concentrations are oxidized at the same rate by brain tissue; that lactate is completely oxidized by brain tissue; that iodoacetic acid (IAA) inhibits glucose oxidation and stated the possibility that glucose necessarily passes through lactic acid for its oxidation to take place; found that oxalate, unlike IAA and NaF, also inhibits the oxidation of lactate.
Holmes, 1933—Hinted at the possibility that lactate oxidation could support brain activity.
Dixon, 1935—Confirmed the fact that in oxygenated brain tissue lactic acid formation cannot be detected, but concluded that the purpose of the complete oxidation of lactate is simply to remove it from the tissue.
Krebs and Johnson, 1937a,b,c; Krebs et al., 1938—Suggested the carbohydrate derivative to enter the Krebs cycle (tricarboxylic acid cycle, TCA) is pyruvate, alas with a question mark.

Nevertheless, even today, more than seven decades after the puzzle of the glycolytic pathway sequence has been resolved, including the identity of its enzymes, substrates and products, if one were to open any of the hundreds of biochemistry textbooks that were published since 1940, glycolysis is described as a process of two separate biochemical pathways. These are described as an aerobic and an anaerobic glycolysis, similar to each other in every enzyme, substrate and product, except for the terminal reaction of the anaerobic one, in which pyruvate is converted to lactate, a conversion catalyzed by lactate dehydrogenase (LDH). Here's a typical description of glycolysis in the fourth edition of *Biochemistry* by Stryer (1995): “*Glycolysis is the sequence of reactions that convert glucose into pyruvate with the concomitant production of a relatively small amount of ATP. In aerobic organisms, glycolysis is the prelude to the citric acid cycle and the electron transport chain, which together harvest most of the energy contained in glucose. Under aerobic conditions, pyruvate enters mitochondria, where it is completely oxidized to CO_2_ and H_2_O. If the supply of oxygen is insufficient, as in actively contracting muscle, pyruvate is converted to lactate*.” This is the dogma that has survived unchanged and mostly unchallenged for all these years. Even in its most recent, seventh edition, *Biochemistry* (Berg et al., 2012) is a textbook that describes glycolysis in somewhat more detail, but unchanged in principles from its 1995 edition. And although one can accept and understand why and how this dogma was developed and formulated with the knowledge that was available in the first half of the twentieth century, the knowledge available today presents several dilemmas that many scientists have chosen to ignore or circumvent due, most probably, to habit of mind (Margolis, 1993). If there is any need for one to realize how strong an influence habit of mind can have, one needs only to recall how successful the, now defunct, lactic acidosis hypothesis of ischemic brain damage had been throughout the 1980s and 1990s (Kalimo et al., 1981; Rehncrona et al., 1981; Siesjö, 1981). Even four decades after the elucidation of the glycolytic pathway it was very easy to persuade a large contingency of scientists who studied possible mechanisms of hypoxic and ischemic brain damage that the culprit behind such damage is no other than the “usual suspect” i.e., lactate.

As has already been mentioned at the beginning of this monograph, Brooks (1985) has demonstrated that lactate is the glycolytic product and the oxidative substrate during sustained exercise. Later, Fox and Raichle (1986) have demonstrated a focal physiological uncoupling between cerebral blood flow and oxidative metabolism upon somatosensory stimulation in humans, and Fox et al. (1988) showed that during focal physiologic neural activity the consumption of glucose is non-oxidative. Simultaneously, Schurr et al. (1988) demonstrated the ability of brain tissue to maintain normal neuronal function with lactate as the sole oxidative energy substrate. With more publications adding support to the possible role of lactate in oxidative energy metabolism, both in muscle (Brooks, 1998, 2000, 2002a,b; Brooks et al., 1999a,b) and especially in brain (Izumi et al., 1994; Pellerin and Magistretti, 1994, 2003; Larrabee, 1995, 1996; Tsacopoulos and Magistretti, 1996; Hu and Wilson, 1997a,b; Schurr et al., 1997a, 1999a,b; Schurr and Rigor, 1998; Magistretti and Pellerin, 1999; Magistretti et al., 1999; Magistretti, 2000; Qu et al., 2000; Van Hall, 2000; Bliss and Sapolsky, 2001; Bouzier-Sore et al., 2003; Mangia et al., 2003; Smith et al., 2003; Dalsgaard et al., 2004; Kasischke et al., 2004; Schurr, 2006; Schurr and Payne, 2007; Herrero-Mendez et al., 2009; Zielke et al., 2009; Schurr and Gozal, 2011), a hot debate has ensued, focusing, unfortunately, on the premise that lactate is somehow an alternative oxidative substrate to glucose in tissue energy metabolism (Chih et al., 2001; Dienel and Hertz, 2001, 2005; Chih and Roberts, 2003; Hertz, 2004; Hertz et al., 2007; Dienel, 2012a,b). Consequently, rather than viewing the oxidative utilization of lactate as an integral part of the oxidative energy metabolic pathway, which begins with glucose and glycolysis and ends with CO_2_, H_2_O and the mitochondrial electron transport chain, many have portrayed lactate as a competitor of glucose. Hence, several studies have aimed at showing that glucose is the obligatory energy substrate for maintenance of various neuronal functions (Dienel and Cruz, 2004; Fillenz, 2005; Bak et al., 2006; Cruz et al., 2007; Gandhi et al., 2009). However, this very role of glucose has never been questioned or challenged by those who unraveled the oxidative utilization of lactate, either by muscle or brain tissue. After all, the principal source of tissue lactate is glucose, a fact that has never been in dispute. The utilization of lactate via its oxidation should have been understood simply as the most plausible and expected progression of glucose breakdown via the glycolytic pathway where lactate, not pyruvate, is the real first step in the mitochondrial TCA cycle. Nonetheless, a concerted effort has been mounted by many established investigators to minimize or marginalize lactate's role in energy metabolism. The following quote from the chapter by Clarke and Sokoloff (1994) on Circulation and Energy Metabolism in the Brain in the fifth edition of Basic Neurochemistry (1994) is most telling: “*Lactate, pyruvate, fructose-1,6-biphosphate, acetate, β-hydroxybutyrate, and acetoacetate can all be utilized by brain slices, homogenates, or cell-free fractions… but the substrate is not available to the brain because of inadequate blood levels or restricted transport through the BBB (blood brain barrier)*.” Clarke and Sokoloff, who were leading scientists in the field of brain energy metabolism at the time, felt compelled to emphasize the limitations lactate faces as an energy substrate specifically in response to the findings of Fox and Raichle (1986), Fox et al. (1988) and Schurr et al. (1988), as if the role of glucose in the process was somehow being diminished by lactate. Hence, Clarke and Sokoloff reemphasize that “*… the nervous system function in the intact animal depends on substrates supplied by the blood and no satisfactory, normal, endogenous substitute for glucose has been found. Glucose must therefore be considered essential for the normal physiological behavior of the central nervous system*.” Therefore, if one requires a proof that habit of mind is long-lived, one could simply follow the heated debate over the role of lactate in oxidative energy metabolism, as exemplified by the large number of con and pro publications on the topic. At least in part, the strong rejection of lactate as an “alternative” oxidative substrate to glucose in energy metabolism, despite the fact that by now this monocarboxylate is recognized as an oxidative substrate, has to do with the old dogma of “lactate is a useless end-product of glycolysis.” Moreover, if lactate, as being offered here, is the real end product of glycolysis, both under aerobic and anaerobic conditions, the established, dogmatic concept of two types of glycolysis would be shaken, rendering the most celebrated pioneering elucidation of a biochemical pathway somewhat misconstrued. Nonetheless, such strong objections, as exhibited by the majority of scientists working in the fields of brain and muscle energy metabolism, to the idea that lactate plays any role and especially a major role in this metabolism, undoubtedly projects the fundamentals of habit of mind (Margolis, 1993), a barrier many find very difficult to cross. This is especially true when some of the most celebrated names in the field are behind the challenged paradigm. Obviously, the aim of this monograph is not to elaborate on the psychology behind habits of mind however, almost all scientists and laymen have accepted the sequence of the glycolytic pathway unchallenged from the time of its inception in 1940. The reasons for such a wide, unchallenged acceptance are beyond the scientific data themselves, reasons that emerged every time a challenge to a long accepted scientific paradigm arose throughout the history of science. I expect that the challenge being put forward here to the dogmatic paradigm depicting glycolysis as two pathways, aerobic and anaerobic, will face as much antagonism as has already being seen in the case of the proposition that lactate plays a role in energy metabolism.

Nonetheless, arguments for a unified, single format of glycolysis, which always ends up with lactate as its final product, are presented below along with arguments against the accepted depiction of two separate types of glycolysis, an aerobic one, with pyruvate as its end product, and an anaerobic one, where lactate is its end product.

## One glycolytic pathway, one starting substrate (glucose), one end product (lactate)

The preceding sections have detailed how and why the pioneers who drew the glycolytic pathway decided to branch it into two types, aerobic and anaerobic. Upon reviewing many of the studies that had led to the formulation of the glycolytic pathway, it becomes clear that the pioneers of that research had faced several hurdles as they put together the available data of their time, including some conflicting results and, understandably, several unknowns. However, glycolysis, as it was drawn then, has remained unchanged today, despite the major dilemmas that the original formulation has created for future generations of investigators in the field of energy metabolism. Tweaking biochemical pathways with the advancement of research is a regular occurrence and yet, the one pathway that has never been tweaked throughout its 70 odd year history is glycolysis. For whatever reason, scientists have been reluctant to suggest any correction or reconsideration of its original formulation. Surely, many would argue that there is absolutely no need for any correction, yet the mere fact that today lactate is an accepted oxidative energy substrate suggests that reconsideration of the original formulation is necessary. Most importantly, maintaining the pathway in its original formulation forces one to circumvent the more straightforward and logical correction i.e., a glycolytic pathway that always ends with the production of lactate, by offering awkward solutions to a major shortcoming of the dogmatic aerobic glycolysis, that is, the need for regeneration of NAD^+^, which assures the maintenance of the cyclical nature of that portion of the pathway. Although this requirement is met in anaerobic glycolysis upon the conversion of pyruvate to lactate and NADH to NAD^+^, this last step does not take place in the formulated aerobic glycolysis, and therefore, aerobic glycolysis, as held today is not capable of regenerating NAD^+^ (the cyclical portion of the pathway that requires lactate production). Although no one knows how or has offered a mechanism by which oxygen converts anaerobic, lactate-producing glycolysis, into an aerobic, pyruvate-producing glycolysis, this process, somehow, has been accepted as an axiom. This is, despite the fact that glycolysis of red blood cells, one of the richest tissues in oxygen concentration, proceeds to produce lactate from glucose in an almost complete stoichiometry, while producing only minute amounts of pyruvate (Bartlett, 1959). No difference is known between the glycolytic apparatus of red blood cells and that of any other tissue and yet, erythrocyte glycolysis produces lactate in the presence of oxygen (or its absence), while glycolysis of any other oxygenated tissue supposedly produces mainly pyruvate. If one could add, in a test tube, mitochondria to oxygenated red blood cells, would glycolysis in these cells produced mainly pyruvate instead of lactate? One could accept for this paradox to remain unresolved until the late waning years of the twentieth century, however, the data accumulated hence forth should be sufficient to explain why this paradox is no more than a misconception. Nevertheless, the scientific community appears content to accept an unresolved paradox, rather than to disturb an imperfect formula of a biochemical pathway.

Since the main problem aerobic glycolysis presents at its original formulation is its inability to reproduce NAD^+^, as pyruvate conversion to lactate via the LDH reaction is supposedly inactive, alternative pathways for NAD^+^ production have been explored.

One such alternative has been offered to be the malate-aspartate shuttle (MAS), a major redox shuttle in brain that supposedly regenerates NAD^+^ when aerobic glycolysis is operational (McKenna et al., 2006; Pardo et al., 2006). Dienel (2012a), in a robust review article, assigns a major role for the MAS in the supply of NAD^+^ to the NAD^+^-less aerobic glycolysis in the brain. The above mentioned review is one of many written by Dienel and colleagues over the years, where they adamantly reject the “suggestion” that lactate could be an alternative oxidative substrate for glucose and hence that glucose is an obligatory energy substrate in the brain. Interestingly, in the above mentioned review Dienel suggests that aerobic utilization of lactate requires a stoichiometric MAS activity to oxidize the NADH in cytoplasm by LDH, and thus, is completely ignoring the possibility that lactate may be oxidized by the mitochondrial LDH and that the NADH thus formed is not in the cytoplasm, but in the mitochondria. The localization of LDH in the mitochondrial membrane and the ability of mitochondria to utilize lactate as a substrate for the TCA cycle have been demonstrated repeatedly (Brandt et al., 1987; Brooks et al., 1999a,b; Brooks, 2002a,b; Hashimoto et al., 2006; Atlante et al., 2007; Schurr and Payne, 2007; Lemire et al., 2008; Passarella et al., 2008; Gallagher et al., 2009; Elustondo et al., 2013; Jacobs et al., 2013). Therefore, the existence of a functional LDH in the mitochondrial membrane negates the need for the MAS as the mechanism responsible for transporting NAD^+^ into the mitochondrion from the cytosol. Acceptance of LDH as a mitochondrial membranous enzyme would, in essence, forces one to question its role there, which is unlikely for the conversion of pyruvate to lactate. Thus, the aggressive push against Brooks et al. (1999a,b) findings, demonstrating the presence of LDH in mitochondrial preparations and its possible role in lactate oxidation was expected (Rasmussen et al., 2002; Sahlin et al., 2002; Ponsot et al., 2005; Yoshida et al., 2007).

**Figure 1:**
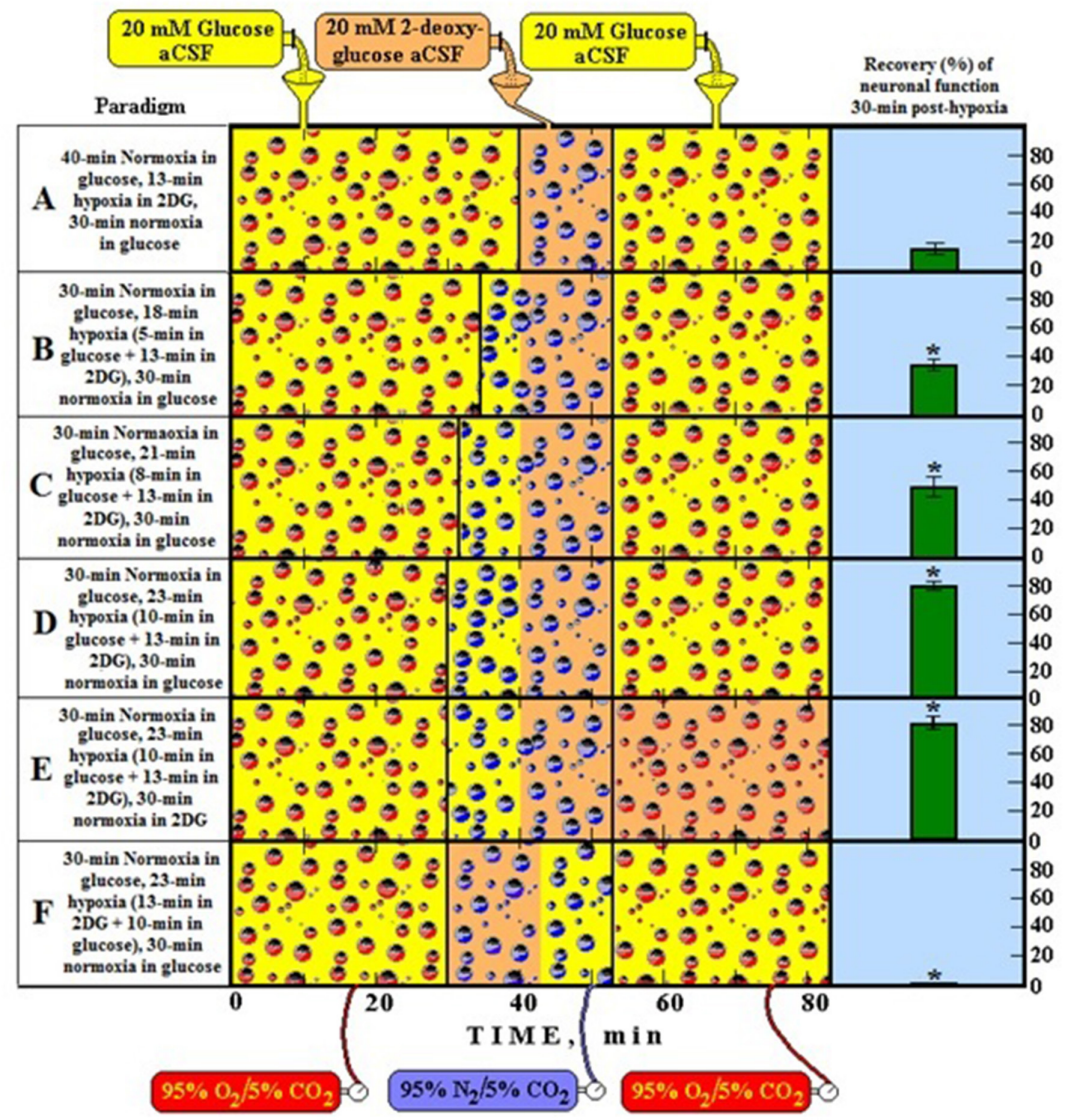
**A schematic representation of six different experimental paradigms using rat hippocampal slices and electrophysiological recording of CA1 evoked population spikes (neuronal function)**. In each experimental paradigm, slices were supplied either with 20 mM glucose (yellow bottle) or 20 mM 2-deoxy glucose (2DG, a glycolytic inhibitor) (orange bottle) and the gas mixture bubbled through the incubation chamber of the slices was either 95% O_2_/5% CO_2_ (normoxia, red bubbles) or 95% N_2_/5% CO_2_ (hypoxia, blue bubbles). At the end of each experimental paradigm, all slices in each compartment of the dual chamber were tested for the presence of neuronal function. Functional slices are shown as percentage of the total number of slices present (green histograms on the right). Accordingly, by following the timeline from left to right, paradigm A is a protocol in which slices were incubated under normoxic conditions for 40 min, followed by 13-min hypoxia in the presence of 2DG and then re-oxygenated for 30 min in the presence of glucose. Under these conditions less than 20% of the slices recovered their neuronal function at the end of the 80-min protocol. Similarly, each of the remaining paradigms (B–F) describes its corresponding protocol and its outcome in terms of percentage of slices exhibiting neuronal function. ^*^Significantly different from paradigm A (*P* < 0.05).

Figure 1 illustrates an *in vitro* experiment (Schurr et al., 1997a) performed using rat hippocampal slices where the cerebral tissue was exposed to hypoxic conditions under which lactate is produced from glucose. Upon reoxygenation, the tissue utilized the hypoxically-produced lactate as the preferred energy substrate over glucose. The more lactate produced during hypoxia prior to 2DG (glycolytic inhibitor) supplementation, the higher the recovery rate of neuronal function posthypoxia (paradigms A–D). When lactate production is blocked during hypoxia, no recovery of neuronal function was observed despite the ample supply of glucose posthypoxia (paradigm F). Even when glucose was not supplied posthypoxia, neuronal function recovered and sustained on hypoxia-produced lactate alone (paradigm E) at least for the duration of the experiment.

**Figure 2:**
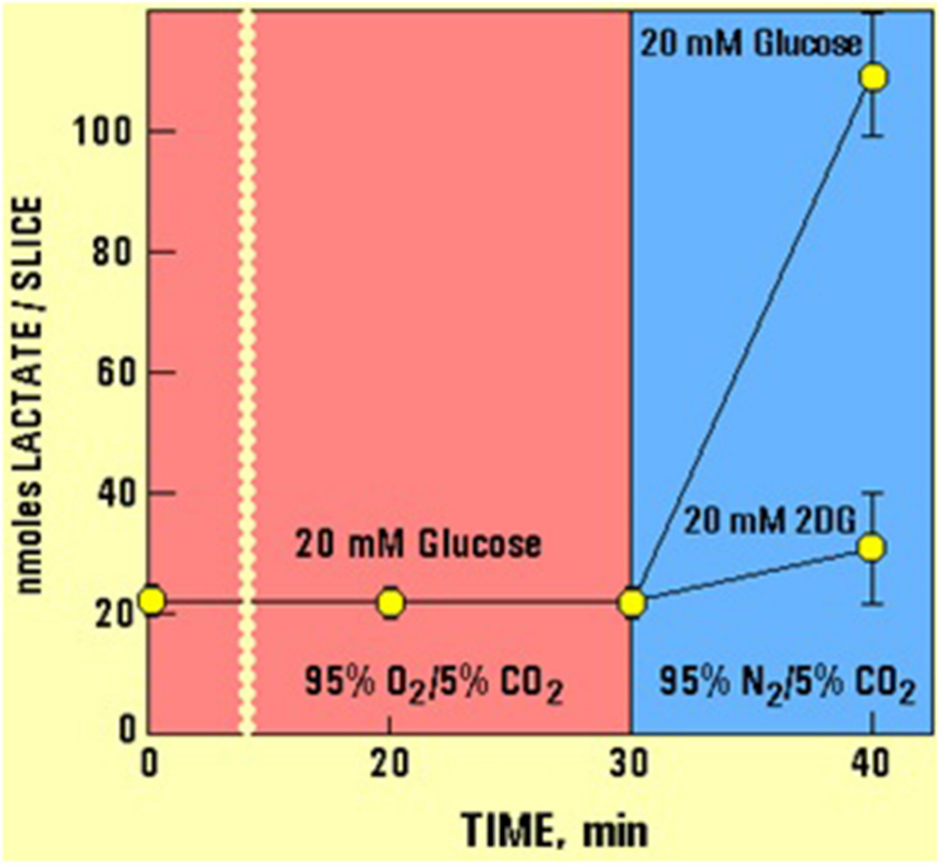
**The levels of lactate and glucose in hippocampal slices (nmoles/slice), as determined using enzymatic kits (Schurr et al., 1997a), during the experimental paradigms D and F detailed in Figure 1**. Allowing slices to utilize glucose anaerobically during the first 10 min of a 23-min hypoxia resulted in an over 5-fold increase in tissue lactate content. Changing the supply of glucose to 2DG at the very beginning of a 23-min hypoxia blocked the ability of hippocampal slices to produce lactate via anaerobic glycolysis.

In a separate experiment (Figure 2, but see also Schurr et al., 1999b) it has been shown that 10 min of hypoxia increased the lactate concentration over 5-fold in hippocampal slices, while 2DG completely blocked that increase.

**Figure 3:**
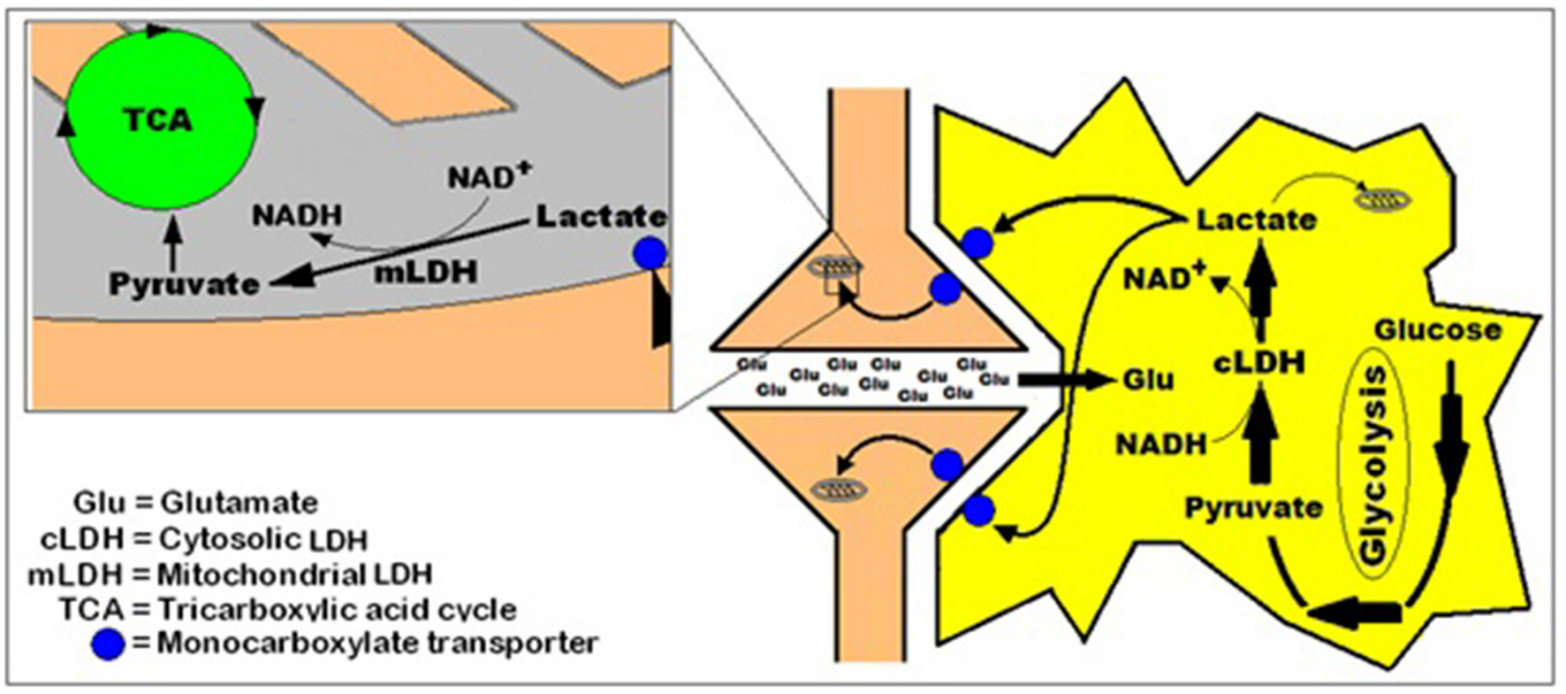
**A simplified schematic representation of the hypothesis of intracellular lactate shuttle between the cytosol and the mitochondria (Brooks, 2000, 2002a,b) and the intercellularly, astrocytic-neuronal lactate shuttle hypothesis (ANLSH, Pellerin and Magistretti, 1994; Herrero-Mendez et al., 2009) combined with the hypothesis that postulates lactate as the end-product of aerobic glycolysis (Schurr, 2006; Schurr and Gozal, 2011)**.

The idea that lactate is shuttled not only intracellularly from the cytosol to the mitochondria, but also intercellularly, as has been shown by many investigations cited above, is illustrated in Figure 3.

Nevertheless, despite the multiple studies that have demonstrated the existence and function of mitochondrial LDH (see above), none of the most recent biochemistry textbooks (Devlin, 2011; Berg et al., 2012) dare mention the presence of LDH in the mitochondrial membrane.

**Figure 4:**
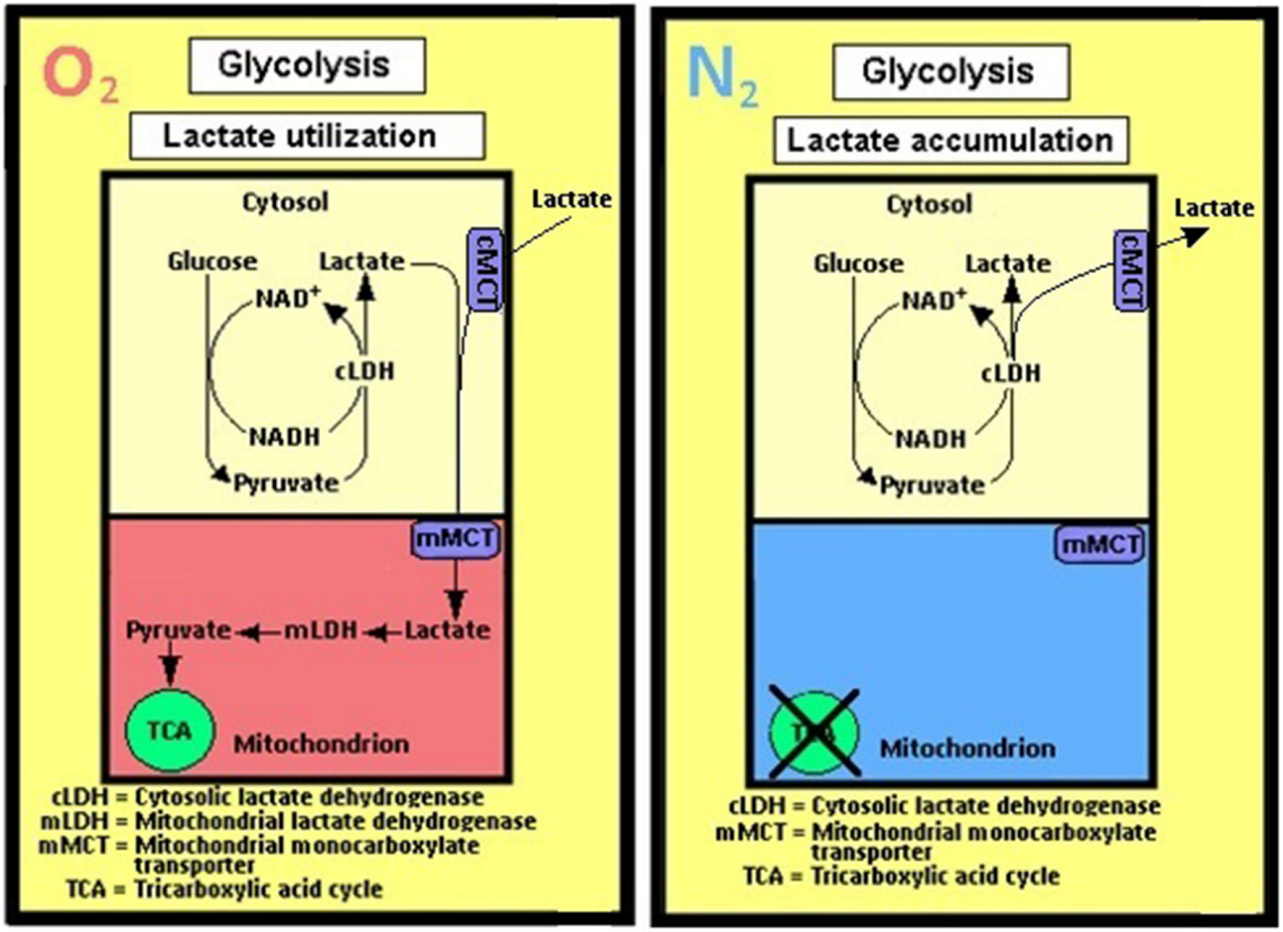
**A schematic representation of the cerebral (and most probably all other tissues) glycolytic pathway as perceived by the author**. Accordingly, no separation is being made between aerobic and anaerobic glycolyses; the singular pathway's first step is the entry of glucose via its phosphorylation to glucose-6-phosphate by hexokinase and the last step is the conversion of pyruvate to lactate by the cytosolic lactate dehydrogenase (cLDH). When mitochondria are functional, in the presence of oxygen (O_2_), lactate is being shuttled from the cytosol to the mitochondrion via the mitochondrial monocarboxylate transporter (mMCT) and, when available, from the extracellular space (Pellerin and Magistretti, 1994; Herrero-Mendez et al., 2009), via the cell membrane monocarboxylate transporter (cMCT). There lactate is oxidized by the mitochondrial lactate dehydrogenase (mLDH) to pyruvate, which enters the tricarboxylic acid (TCA) cycle, hence lactate utilization. The only difference between glycolysis under oxygen (O_2_) atmosphere and glycolysis under nitrogen (N_2_) atmosphere is lactate accumulation and release into the extracellular space under the latter, as it cannot be oxidized in the mitochondria.

With the abundance of published studies over the past 39 years, all pointing, in one way or another, at a simpler, straight forward, singular glycolytic pathway, it is of utmost importance to redefine “glycolysis” as the cytosolic biochemical pathway that under both, aerobic and anaerobic conditions, constantly utilizes glucose as its initial substrate to produce lactate as its end product in cerebral tissue and in almost any other tissue. The NAD^+^ that is reduced to NADH during the formation of pyruvate is being reformed by the glycolytic LDH during the conversion of pyruvate to lactate, affording this portion of the pathway its cyclical nature. Under aerobic conditions lactate is utilized as the main substrate of the mitochondrial tricarboxylic acid (TCA) cycle, and thus, is defined as the major coupler between the two pathways, each localized in a separate cellular compartment, the cytosol and the mitochondrion, respectively. In the mitochondrion, lactate, transported from the cytosol via a monocarboxylate transporter (MCT) (Mowbray, 1975; Brooks et al., 1999a), is oxidized to pyruvate by the mitochondrial LDH (mLDH), which also provides the mitochondrion with NADH, circumventing the need for the proposed function of the malate-aspartate shuttle (MAS). Under anaerobic conditions, glycolysis continues to function unabated, resulting in lactate accumulation, as the TCA cycle is non-functional (Figure 4). When lactate is accumulating, under anaerobic conditions, it becomes, upon return to aerobic conditions, the principal energy substrate until its levels are falling back to their minimal normal levels (Figure 1, Schurr et al., 1997a,b, 1999a).

## Summary

Lactate is a metabolite that has earned a negative reputation ever since its discovery 234 years ago. Therefore, with the advent of biochemistry and the elucidation of the different pathways involved in carbohydrate metabolism and bioenergetics, any appearance of lactate seemed to signal harmful or damaging consequence, such that any reaction or treatment that could minimize lactate concentration was considered beneficial. The large scientific community that worked on muscular carbohydrate metabolism had determined the tone and direction of their research and influenced how the research of carbohydrate metabolism in other tissues, especially brain, was interpreted. Hence, when studies in the mid-1980s have appeared to challenge the prevailing dogma, assigning a possible role, perhaps even a crucial role, for lactate in energy metabolism, the habit of mind of the majority within the scientific community then, and even today, erected a barrier that prevents the acceptance of such role, despite the continuously growing number of publications in support of it. This monograph attempted to review the publications of the first four or five decades of muscle and brain carbohydrate metabolism in an effort to persuade its readers to open their minds to the possibility that glycolysis, in cerebral and other tissues, is a singular pathway, in the presence or absence of oxygen, which begins with glucose as its substrate and terminates with the production of lactate as its main end product.

### Conflict of interest statement

The author declares that the research was conducted in the absence of any commercial or financial relationships that could be construed as a potential conflict of interest.
